# Immunodepletion of amyloid‐associated matrisomal proteins reduces Aβ in hiPSC‐derived neurons

**DOI:** 10.1002/alz.084611

**Published:** 2025-01-03

**Authors:** Benjamin Siciliano, Qianjin Li, Riham Ayoubi, Carl Laflamme, Elizabeth Zoeller, Yuhong Du, Haian Fu, Zhexing Wen, Allan I. Levey, Ranjita Betarbet

**Affiliations:** ^1^ Emory University, Atlanta, GA USA; ^2^ McGill University, Montreal, QC Canada; ^3^ Emory University School of Medicine, Atlanta, GA USA

## Abstract

**Background:**

Large‐scale unbiased proteomic profiling studies have identified a cluster of 31 proteins co‐expressed with APP, which is termed the matrisome module 42 (M42). M42 is enriched in AD risk genes, including APOE, with mostly secreted proteins that bind heparin, collectively strongly correlate with the burden of brain pathology and cognitive trajectory, and localize to amyloid plaques in AD brain. For these reasons, M42 has been nominated as a novel therapeutic target for enabling drug discovery by our TREAT‐AD Center. Here we immunodeplete several M42 proteins including MDK, SFRP1, HTRA1, QPRT, PTN, and SMOC1 using iPSC‐derived neurons modeling autosomal dominant AD to investigate effects on amyloid production.

**Method:**

The AD‐related APP Swedish mutation (APP KM670/671NL) was introduced into the well‐characterized wildtype control iPSC line (WTC‐11), and programming by the transcription factor neurogenin‐2 (NGN2) was used to generate cultures of high purity excitatory neurons. Inhibition of a collection of M42 proteins with strong correlation with AD endophenotypes was performed by treating neurons with recombinant monoclonal antibodies. Western blot was used to confirm immunodepletion of the secreted protein targets, as well as to measure p‐Tau and total Tau. Enzyme‐linked immunosorbent assay (ELISA) kits specific for human Aβ40 and Aβ42 were used to quantify Aβ species.

**Result:**

Aβ40, Aβ42, p‐Tau (AT8), p‐Tau (S396), and total Tau, as well as ECM‐associated proteins (MDK, SFRP1, HTRA1, QPRT, PTN, SMOC1) were elevated in neuronal media and cell lysate derived from hiPSCs carrying the APP Swedish mutation relative to WT controls. Immunodepletion of MDK, HTRA1, and PTN reduced Aβ species in neuronal media, while immunodepletion of QPRT increased Aβ species and SFRP1 showed no significant changes.

**Conclusion:**

hiPSCs with the APP Swedish mutation express and secrete increased levels of several M42 amyloid‐associated proteins as in human AD. Reductions in Aβ species following immunodepletion of MDK, HTRA1, and PTN demonstrate the utility of hiPSC models for probing M42 function and therapeutic potential for AD.

